# Iodide of Zinc

**Published:** 1854-01

**Authors:** 


*Iodide of Zinc.*—During the last six months an extended series of trials of a new drug, the iodide of zinc, has been made in Guy's Hospital on patients under the charge of Dr. Barlow. The salt has been prepared, we believe to Dr. Barlow's direction, by Messrs. Davenport, and is given in the form of a syrup, of which the dose is the same as of iodide of iron. Dr. Barlow considers it especially indicated in cases in which the zinc alone might be too stimulating, and in those in which it is desirable to have the effects of both the ingredients. Chorea, struma, cachexia, and some forms of hysteria, form the chief in which it has been tried. In one case of severe strumous superficial lupus, it has been continued for many months and in full doses, with great apparent benefit. It is doubtful whether the cases of chorea have improved more rapidly than they might have been expected to do under the usual treatment by the sulphate.—*Med. Times and Gazette.*

